# Community Knowledge and Experience of Mosquitoes and Personal Prevention and Control Practices in Lhasa, Tibet

**DOI:** 10.3390/ijerph110909919

**Published:** 2014-09-23

**Authors:** Xiaobo Liu, Fangjun Wan, Cirendunzhu, Cirenwangla, Li Bai, Pengcuociren, Lin Zhou, Baimaciwang, Yuhong Guo, Dazhen, Junfang Xu, Shaowei Sang, Xiaolu Li, Shaohua Gu, Haixia Wu, Jun Wang, Dawa, Xiraoruodeng, Qiyong Liu

**Affiliations:** 1 State Key Laboratory for Infectious Disease Prevention and Control, National Institute for Communicable Disease Control and Prevention, Chinese Center for Disease Control and Prevention, 155 Changbai Road, Changping District, Beijing 102206, China; E-Mails: liuxiaobo@icdc.cn (X.L.); wanfangjun1989@163.com (F.W.); yan_lili407@126.com (L.B.); zhoulin.scu@163.com (L.Z.); guoyuhong@icdc.cn (Y.G.); sangshaowei1@163.com (S.S.); gushaohua1989@sina.com (S.G.); wuhaixia@icdc.cn (H. W.); wangjun@icdc.cn (J.W.); 2 WHO Collaborating Centre for Vector Surveillance and Management, 155 Changbai Road, Changping District, Beijing 102206, China; 3 China CDC Key Laboratory of Surveillance and Early-Warning on Infectious Disease, 155 Changbai Road, Changping District, Beijing 102206, China; 4 Tibet Center for Disease Control and Prevention, 21 Linkuo North Road, Lhasa 850000, China; E-Mails: cirendunzhuok@126.com (C.); pc911amcdc@163.com (P.); nc916@126.com (B.); dazhen199478@163.com (D.); xzcdcdawa@sina.com (D.); xzcdc-xr@163.com (X.); 5 Center for Health Management and Policy, Shandong University, 44 Wenhua Xi Road, Jinan 250014, Shandong, China; E-Mails: junfangxuhappy1987@163.com (J.X.); xiaoluligood@163.com (X.L.); 6 Collaborative Innovation Center for Diagnosis and Treatment of Infectious Diseases, Hangzhou 310003, China; 7 Lhasa Chengguan District Center for Disease Control and Prevention, 1 Hongqi West Road, Lhasa 850000, China; E-Mail: 18908916466@189.cn

**Keywords:** knowledge, experience, prevention and control practice, mosquito, public health, Tibet

## Abstract

Since 2009, great public attention has been paid in Lhasa City (Tibet, China) to mosquito bites and accompanying inflammatory complications. However, the potential contribution of knowledge levels, experiences, disease control and preventive practices (KEP) towards mosquitoes has not received much attention. To investigate community KEP concerning mosquitoes in Lhasa, a cross-sectional survey was undertaken in four sub-districts of urban Lhasa in 2012. Questionnaires were designed to collect information regarding socio-demographics and KEP concerning the harmful effects of mosquitoes on participants. The scoring for KEP was developed after consultation of literature. A total of 591 eligible questionnaires were examined. The majority of respondents were female (61.8%) with a mean age of 46 years. Nearly all of the respondents were of Tibetan nationality (97.4%) and living in registered native households (92.7%), who have less than primary school education. The averages of overall score, knowledge score, experience score, and practice score were 9.23, 4.53, 1.80, 2.90, respectively. The registered household with the highest overall score, knowledge score and practice score was non-native. Female subjects with monthly incomes between 1000 and 3000 RMB had higher experience scores. The correlation analysis revealed that significant positive linear correlations existed between knowledge and experience, knowledge and practices, and experience and practices towards mosquitoes. Past experiences with mosquitoes can result in a better knowledge of effective mosquito control practices in the present and the future. Though the average of overall scores related to mosquitoes is high among the participants in Lhasa, however, the knowledge about the ecological habits of mosquitoes should be strengthened. The findings in this study may help to develop strategies and measures of mosquito and mosquito-borne diseases in the future, not only in Lhasa, but also in similar altitude, latitude and longitude regions worldwide.

## 1. Introduction

Mosquito-borne diseases and pestiferous mosquito species are a major public health problem worldwide [1]. Diseases such as West Nile disease, malaria, dengue fever and Chikungunya fever are undergoing a resurgence and redistribution under global climate change [2,3,4]. Mosquito-borne diseases are now being reported at high elevations in the highlands of Asia, Central Africa, and Latin America, and malaria could threaten major elevated urban centers such as Nairobi, Kenya [5]. In China, mosquito-borne diseases continue to be a serious public health problem and have caused substantial morbidity and mortality in recent years [6].

Lhasa City is the administrative capital of the Tibet Autonomous Region (TAR) of China. The average altitude of Lhasa is about 3650 m above sea level, with a 2010 population of 559,423 people. The climate is of the temperate plateau monsoon type. With an annual average temperature of 7.5 °C, Lhasa has an annual precipitation of 426 millimeters, with rain falling mainly from July to September. In 2009, reports appeared relating to the emergence of mosquitoes in urban Lhasa. Our previously study has confirmed that the local *Culex pipiens* complex comprised *Cx. pipiens quinquefasciatus*, *Cx. pipiens pipens*, *Cx. pipiens pallens*, and its hybrids [7]. In China, numerous studies have shown that mosquitoes of the *Cx. pipiens* complex tend to be anthropophilous rather than livestock or poultry feeders [8]. Once established in a high altitude region, the subspecies in the *Cx. pipiens* complex, may threaten the health of humans and vertebrates due to their ability to transmit numerous diseases, such as the West Nile virus [9,10,11,12], filariasis [13,14,15,16], Japanese encephalitis, St. Louis encephalitis [17,18], and avian malaria (*Plasmodium* spp*.*) [13,18]. With global warming, the risk of mosquito incursions and spread will be more prevalent at high altitude cities [6,19].

Exploratory studies of the knowledge, experience, and prevention and control practice (KEP practice) concerning mosquitoes in a region, are of great significance for developing community-based strategies and measures for the control and prevention of mosquito-borne diseases [1]. In essence, the KEP questionnaire survey related to mosquitoes is similar to the knowledge, attitude, and practice (KAP) survey. Based on literature review, the KAP survey’s purpose is to collect information on what is known, believed and done within a specific representative population in relation to a particular issue. Typical questions of knowledge include causes, symptoms of the illness under investigation, *etc.* In upstate New York, knowledge of WNV was measured by asking questions about mosquito vectors, common infected animals, disease symptoms, risk groups and preventive measures [20]. Attitude has been defined as “a learned predisposition to think, feel and act in a particular way towards a given object or class of objects” [21,22,23,24,25,26,27,28,29,30]. Experience refers to a particular instance of personally encountering something or the process of personally observing or undergoing something [31]. Mosquito experience refers to the process of personally encountering, observing, or being bitten by mosquitoes in restricted environments in the past. Because mosquitoes became established in Lhasa City in recent years, therefore, the investigation of the residents’ experience of mosquitoes can reflect the proportion of native people exposed to mosquitoes, and can also provide guidance for the prevention and control of mosquitoes and potential mosquito-borne diseases in the future. On the contrary, the proportion of exposure cannot be obtained by attitude investigation. Practice usually enquires about the use of preventive measures [32]. Practices surveys related to mosquito or mosquito-borne diseases usually enquire about the use of preventive measures or different health care options.

Good knowledge, experience and practices (KEP) among the public regarding mosquito control prevention is required for the success of disease control and the protection of the susceptible population [33,34,35,36]. However, very little is known about mosquito control and prevention KEP among the general population in Lhasa. The objective of this study was to investigate the community’s KEP concerning mosquitoes in four urban sub-districts in Lhasa. The findings may serve to develop targeted strategies and measures applicable to the *Cx. pipiens* complex and its related mosquito-borne diseases in the future, not only in Lhasa, but also in other geographically similar regions.

## 2. Methods

### 2.1. Study Area

**Figure 1:**
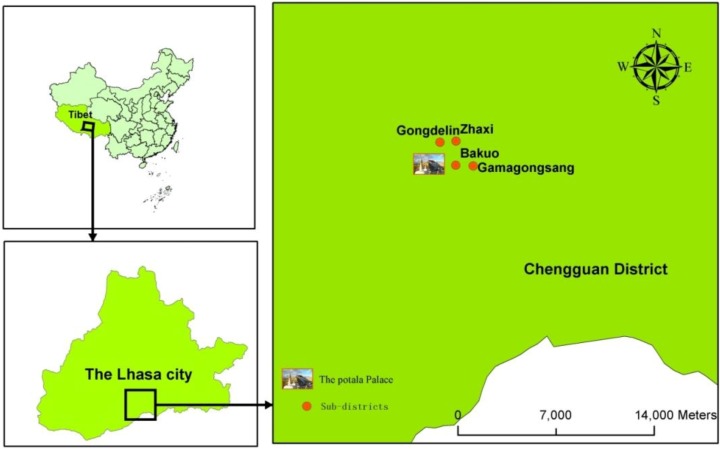
The four urban sub-districts of this study.

A cross-sectional questionnaire survey was conducted in the urban Chengguan district which is the only municipal district in Lhasa City, from 16 to 19 August in 2012. This district was selected because it is representative of the local population (accounting for nearly 50% of the total population of Lhasa City). This district has a total population of 279,074, with an area of 554 square kilometers and an average elevation of 3658 m. Four urban sub-districts (Gongdelin, Gamagongsang, Zhaxi and Bakuo) (Figure 1) were selected from all six urban sub-districts of Chengguan District to ensure a good geographical coverage of urban Lhasa and obtain data that best represents the diverse demographic and socio-economic characteristics of its population [37]. These four sub-districts are composed of 20 neighborhoods and the socio-demographic characteristics of neighborhoods within the same sub-districts are comparable. The questionnaire survey was carried out in the 20 neighborhood committees which are located at the centers of each neighborhood.

Gongdelin lies to the northwest of the Potala Palace and adjacent to the “Lalu Wetland”; it has a population of 31,492 and an area of 4.1 km^2^ (population density: 7757 people/km^2^). Residents are relatively wealthy and mainly live in self-built single family houses with a small yard. Gamagongsang lies to the east of the Potala Palace with a population of 26,165 and an area of 3.2 km^2^ (population density: 8177 people/km^2^). There is limited infrastructure such as drainage systems and roads, and there are no parks or other urban green spaces. Zhaxi lies to the northeast of the Potala Palace and adjacent to the “Lalu Wetland” with a population of 44,775 and an area of 6.0 km^2^ (population density: 7462 people/km^2^). The development level of this district is similar to that of Gamagongsang. Compared with Gamagongsang, there is more open and green public space in Zhaxi. Bakuo lies to the northeast of the Potala Palace and adjacent to the “Lalu Wetland” with a population of 24,650 and an area of 2.7 km^2^ (population density: 9129 people/km^2^). Bakuo is in the business center of the urban core. It is the most popular, noisy and crowded place in urban Lhasa. Residents appear to have low educational level, low income, small houses and poor dwelling conditions.

### 2.2. Study Sample Size and Sampling Procedure

By employing a stratified random sampling method, approximately 600 respondents were targeted for the study. Because no data was available concerning KEP survey of mosquitoes in Lhasa in the past, therefore, this sample size was determined by literature review and the sample size was deemed adequate to clarify the true level of neighborhood knowledge, experience and practices towards mosquitoes [1]. Because the population density of the selected sub-districts was similar, 150 people within each sub-district were investigated. Surveys were administered to residents older than 18 years who lived in the four urban sub-districts all year around. Residents who is younger than 18 years or lived in other urban sub-districts were excluded in this study.

### 2.3. Data Collection

To collect information regarding socio-demographics and KEP participants, a questionnaire was developed by referring to relevant literatures [21,38,39]. Prior to the field investigation, we pre-tested the questionnaire with a small scale pilot study in Lhasa, which included potential data collectors and administrators. The questionnaire was revised for assessing content validity, appropriateness and question comprehensibility. A face-to-face interview was adopted. Data collectors and questionnaire administrators were staff members from the National Institute for Communicable Disease Control and Prevention (ICDC), China CDC and Tibet CDC. The survey administration was taken place in the specific urban sub-district of Lhasa. Some Tibetans were recruited to serve as interpreters. In Tibet CDC, one-day training was delivered to the surveyors prior to field investigation. Incomplete questionnaires were returned to surveyors for its completion by a telephonic interview.

The socio-demographic information collected in this questionnaire included: age, gender, ethnicity, household register, education, monthly income and occupation of the respondents, among others. In addition to the socio-demographic data, the questionnaire included: six questions that explored knowledge related to mosquitoes (heard of mosquitoes, access to mosquito related knowledge, mosquito breeding sites, mosquito blood-sucking habits, transmission diseases, and the impact of mosquitoes on people’ daily life); two questions focused on participant’s experience with mosquitoes (encountered mosquitoes, bitten by mosquitoes); and four questions which addressed control and prevention practices towards mosquitoes (the necessity for prevention and control of mosquitoes, strategies and measures towards control and prevention, accessibility to anti-mosquito products, and suggestions concerning mosquito control and prevention).

As to specific access to mosquito-related knowledge, we also set multiple choices. These answers included interpersonal communication, internet and television, books, literatures, monographs, neighborhood committees, bulletin boards, brochures, not pay attention to, no good channels, *etc.* Regarding the bitten by mosquitoes question, we set up more questions if the respondent was bitten by mosquitoes prior to this investigation. These questions related to the months and years that most of these bites occurred, the frequency of being bitten by mosquitoes in these months, and the status of hospitalization for any serious complications. Concerning the assessment of prevention and control practice of mosquito we also set some prevention and control measures, such as mosquito coils, door and window screens, repellent products, long sleeved clothes, mosquito-killing aerosols, swatters, stagnant water elimination, mosquito nets, light traps, and Tibetan incense. Regarding access to acquire mosquito repellent products, we also set some options. These mainly included supermarkets, grocery stores and small market stalls.

The scoring mechanism for knowledge, experience, control and prevention practice (KEP) was developed by the researchers inspired by some literature models. Each KEP question was assessed by giving one point to a correct or positive answer and zero points to incorrect or negative answers [1,21,40]. Each question only had one correct or positive answer. The final KEP score was calculated by summing up the scores of knowledge, experience and control and prevention practices sections individually [20,40]. In this study, the scores of each respondents were not classified into poor, average, and good because of the relatively fewer questions.

### 2.4. Data Analysis

Descriptive statistics were utilized to illustrate the socio-demographic characteristics of the respondents. Categorical variables were measured as percentages and continuous variables were expressed as Mean ± Standard Deviation (SD). The overall score, knowledge score, experience score, and practice score of each respondent and all respondents were calculated. Because scores presented for the KEP questionnaire exhibit distinct maximum values for each one of its components, therefore, the results of each parameter were standardized by Z-score method prior to a direct comparison. Because general linear models (GLMs) are generally robust against most violations of parametric assumptions (normality and homogeneity of variances), therefore, univariate analysis of variance (general linear models) was used to examine the relationship between background and knowledge score, experience score, practice score, and the overall score. Pearson correlation analysis was adopted to clarify the correlation between knowledge, experience, prevention and control practice towards mosquitoes [21]. Probabilities of the univariate analysis of variance were at a = 0.05 level (SPSS version 17.0; SPSS Inc., Chicago, IL, USA).

### 2.5. Ethical Approval

We obtained ethical approval from the Ethical Review Committee of Chinese Center for Disease Control and Prevention (No.201214). Written informed consent was obtained from all the respondents prior to survey administration. Permission was also obtained from the directors of Tibet CDC and the participant neighborhoods in Lhasa city.

## 3. Results and Discussion

### 3.1. Socio-Demographic Characteristics

The identified socio-demographic characteristics of the 591 eligible participants in Lhasa (a total of 629 respondents were interviewed) are shown in Table 1. Most of the interviewees were females (61.8%) of Tibetan nationality (97.4%) and living in a registered native household (92.7%), with a lower than primary school education (59.5%) and low income (<1000 RMB/month, 45%). The average age of the participants was 46.36 (SD = 15.23) year (range 18~86 yr) with the great majority of participants (426, 72.0%) being between 30 and 65 years old.

**Table 1:** Socio-Demographic characteristics of the study participants (*N* = 591) in urban Lhasa City, 2012.

Variables	Category	Number of Interviewees	Proportion within Each Category (%)
Study sites	Gongdelin	148	25.0
	Gamagongsang	153	25.9
	Zhaxi	125	21.2
	Bakuo	165	27.9
Gender	Male	226	38.2
	Female	365	61.8
Ethnic group	Tibetan	575	97.4
	Hui	2	0.3
	Han	12	2.0
	Others	2	0.3
Register household	Native	548	92.7
	Non-native	43	7.3
Age (y)	18~	90	15.3
	30~	151	25.5
	42~	149	25.2
	54~	126	21.3
	65~	75	12.7
Education	Illiteracy	138	23.4
	Literacy class	7	1.2
	Primary school	206	34.9
	Middle school	90	15.2
	High school	100	16.8
	Junior college and above	50	8.5
Monthly income (*RMB*)	Less than 1000	266	45.0
	1000~	167	28.3
	2000~	79	13.4
	3000~	35	5.9
	4000~	23	3.9
	Higher than 5000	21	3.5
Occupation	Worker	8	1.4
	Migrant laborer	58	9.8
	Peasant	10	1.7
	Student	18	3.0
	Cadre and office worker	107	18.1
	Chauffeur	8	1.4
	Teacher	8	1.4
	Medical personnel	4	0.7
	Retired employee	72	12.2
	Catering Service staff	1	0.2
	Self-Employed	35	5.9
	Homemaker	23	3.9
	Unemployed youth	215	36.2
	Unwilling to answer	21	3.6
	Other	3	0.5

### 3.2. Assessment of Mosquito Knowledge

The correct or positive rate of responses to knowledge, experience, prevention and control practice items concerning mosquito is shown in Table 2. In terms of knowledge, among the 578 respondents who have heard about mosquitoes, 73.9% have access channels to mosquito-related knowledge (the primary source was through interpersonal communication, then internet and television); 71.8% of participants know that mosquitoes can suck human blood; 88.9% believed mosquitoes can affect daily life. Unfortunately, only 40.5% of participants knew that mosquitoes could be found in a variety of water bodies.

**Table 2:** Responses to mosquito knowledge, experience and practice items.

Items *	Variables	Category	Frequency (%)
Knowledge	*Have you heard of mosquitoes prior to this investigation?*	Yes	578 (97.8%)
		No	13 (2.2%)
	*Do you have access channels to acquire mosquito-related knowledge?*	Yes	427 (73.9%)
		No	151 (26.1%)
	*Can mosquitoes be found in a variety of water bodies?*	Yes	234 (40.5%)
		No	344 (59.5%)
	*Can mosquitoes suck human or animal blood?*	Yes	415 (71.8%)
		No	163 (28.2%)
	*Can mosquitoes transmit some diseases?*	Yes	450 (77.9%)
		No	128 (22.1%)
	*Can mosquitoes exert some impacts on people* *’* *s daily life?*	Yes	514 (88.9%)
		No	64 (11.1%)
Experience	*Have you ever seen mosquitoes in Lhasa?*	Yes	546 (94.5%)
		No	32 (5.5%)
	*Have you ever been bitten by mosquitoes in Lhasa?*	Yes	493 (85.3%)
		No	85 (14.7%)
Practice	*Is it necessary to control and prevent mosquitoes in summer?*	Yes	531 (91.9%)
		No	47 (8.1%)
	*Have you ever taken some mosquito control products and measures?*	Yes	446 (77.2%)
		No	132 (22.8%)
	*Is it convenient to purchase mosquito repellent products?*	Yes	507 (87.7%)
		No	71 (12.3%)
	*Can you give some suggestions to government and stakeholders concerning mosquito control in future?*	Yes	193 (33.4%)
		No	385 (66.6%)

* Knowledge, experience, and practices were assessed by giving 1 point to the correct answer and 0 point to the wrong answer.

The mean knowledge score for the entire study was 4.53 (SD = 1.01). There were noticeable differences in the average knowledge score among registered households (*P* *<* 0.05). The registered household with a relatively higher knowledge score was non-native (Table 3).

### 3.3. Assessment of Experience with Mosquitoes

In the current study, 94.5% (546) of respondents who had heard about mosquitoes had seen mosquitoes in different locations, and 85.3% (493) of them were bitten by a mosquito (Table 2). Approximately 60% of respondents confirmed that most of these bites occurred after 2009, specifically in 2012 (44.9%). As to seasonal distribution, 50.3% residents recalled mosquitoes could be mostly observed in July and 59.8% residents mentioned they were bitten by mosquitoes more than three times per month. Up to 4.5% of respondents required hospitalization for serious complications. The mean experience score was 1.80 (SD = 0.51) (Table 3). There were obvious differences in the score of the average experience in terms of gender (*P* *<* 0.05) and monthly income (*P* *<* 0.01). Females, with a monthly income between 1000 and 3000 RMB*,* had the relatively higher score on experience.

### 3.4. Assessment of Prevention and Control Practices of Mosquito

As to the prevention and control practices, 531 (91.9%) respondents declared it was necessary to control mosquitoes during the summer. Unfortunately, only 446 (77.2%) respondents had applied measures to control mosquito population (Table 2). In this study, the prevention and control measures were ranked depending on utilization. Mosquito coils were used by 50.4% of the respondents, door and window screens 49.3%, repellent products 41.9%, long sleeve clothes 41.0%, mosquito-killing aerosols 21.5%, swatters 14.8%, stagnant water elimination 9.4%, mosquito nets 4.3%, light traps 2.9%, Tibetan incense 2.0% and others 4.3%.

Roughly 87.7% (507/578) of respondents believed it is convenient to acquire mosquito repellent products. The main purchase channels were supermarkets (83.2%), grocery stores (28.5%) and small market stalls (21.1%). Only 193 (33.4%) respondents gave suggestions to stakeholders concerning mosquito control, including increased supply of insecticide products, environmental protection, timely disposal of garbage, hygiene improvements, and advertising of anti-mosquito efforts.

The mean practice score for the entire study was 2.90 ± 0.92 (Table 3). There were discernible differences in the households’ average practices (*P* *<* 0.05) scores. Non-native households had relatively higher practice scores concerning mosquitoes than native households.

**Table 3:** Comparison of demographic characteristics and mean KEP scores.

Variable		N (578 )	Overall Score (M ± D)	*P*	Knowledge Score (M ± D)	*P*	Experience Score (M ± D)	*P*	Practice Score (M ± D)	*P*
Study sites	Gongdelin	147	9.068 (1.777)	0.123	4.41 (0.985)	0.356	1.73 (0.568)	0.169	2.93 (0.951)	0.802
	Gamagongsang	149	9.322 (1.713)		4.52 (1.011)		1.87 (0.445)		2.94 (0.932)	
	Zhaxi	121	9.388 (1.614)		4.64 (1.080)		1.78 (0.524)		2.98 (0.790)	
	Bakuo	161	9.167 (1.754)		4.57 (0.980)		1.81 (0.503)		2.79 (0.977)	
Gender	Male	218	9.262 (1.635)	0.474	4.48 (1.048)	0.459	1.78 (0.505)	0.040	3.00 (0.880)	0.108
	Female	360	9.208 (1.773)		4.56 (0.988)		1.81 (0.517)		2.84 (0.944)	
Nationality	Tibetan	562	9.228 (1.736)	0.619	4.54 (1.011)	0.578	1.80 (0.511)	0.919	2.89 (0.930)	0.758
	Hui	2	9.500 (0.707)		4.50 (0.707)		2.00 (0.000)		3.00 (0.000)	
	Han	12	9.250 (1.288)		4.33 (1.155)		1.75 (0.622)		3.17 (0.577)	
	others	2	9.000 (0.000)		4.00 (0.000)		1.50 (0.707)		3.50 (0.707)	
Household register	Native	540	9.228 (1.722)	0.002	4.52 (1.002)	0.046	1.81 (0.490)	0.731	2.89 (0.929)	0.021
	Non-native	38	9.237 (1.731)		4.68 (1.141)		1.55 (0.724)		3.00 (0.838)	
Age group (y)	18~	88	9.386 (1.441)	0.148	4.60 (0.989)	0.728	1.87 (0.424)	0.113	2.91 (0.879)	0.390
	30~	147	9.456 (1.627)		4.64 (0.986)		1.84 (0.454)		2.98 (0.910)	
	42~	147	9.150 (1.741)		4.52 (0.995)		1.80 (0.548)		2.83 (0.894)	
	54~	122	9.254 (1.770)		4.57 (0.996)		1.79 (0.502)		2.90 (0.991)	
	65~	74	8.702 (1.991)		4.18 (1.090)		1.65 (0.629)		2.88 (0.950)	
Education	Illiteracy	130	9.085 (2.004)	0.343	4.47 (1.136)	0.940	1.78 (0.517)	0.429	2.84 (0.963)	0.512
	Literacy class	7	9.143 (1.773)		4.71 (1.113)		1.86 (0.378)		2.57 (0.787)	
	Primary school	203	9.123 (1.752)		4.51 (0.982)		1.77 (0.563)		2.84 (0.977)	
	Middle school	89	9.202 (1.740)		4.48 (1.035)		1.80 (0.526)		2.92 (0.956)	
	High school	99	9.586 (1.385)		4.70 (0.974)		1.84 (0.445)		3.05 (0.761)	
	Junior college and above	50	9.380 (1.276)		4.48 (0.789)		1.88 (0.385)		3.02 (0.820)	
Monthly income (RMB)	Less than 1000	260	9.135(1.875)	0.083	4.51 (1.067)	0.250	1.78 (0.541)	0.009	2.84 (0.956)	0.287
	1000~	164	9.433 (1.551)		4.68 (0.884)		1.82 (0.495)		2.93 (0.897)	
	2000~	79	9.215 (1.550)		4.42 (1.057)		1.85 (0.455)		2.95 (0.830)	
	3000~	34	9.235 (1.372)		4.29 (0.760)		1.85 (1.359)		3.09 (0.965)	
	4000~	22	8.546 (2.110)		4.18 (1.296)		1.50 (0.598)		2.86 (1.125)	
	High than 5000	19	9.579 (1.539)		4.79 (0.918)		1.79 (0.535)		3.00 (0.745)	
Occupation	Labour	8	9.500 (1.309)	0.204	4.50 (1.195)	0.383	1.75 (0.463)	0.924	3.25 (0.463)	0.217
	Migrant labourer	57	9.263 (1.541)		4.35 (1.009)		1.82 (0.504)		3.09 (0.892)	
	Peasant	10	8.700 (1.703)		4.00 (1.333)		2.00 (0.000)		2.70 (1.160)	
	Student	18	9.611 (1.754)		4.56 (1.338)		1.89 (0.471)		3.17 (0.786)	
	Cadre and office	106	9.528 (1.148)		4.63 (0.797)		1.86 (0.424)		3.04 (0.780)	
	Chauffeur	7	9.143 (1.952)		4.29 (1.113)		2.00 (0.000)		2.86 (0.900)	
	Teacher	8	9.875 (0.834)		4.25 (0.463)		2.00 (0.000)		3.63 (0.518)	
	Medical personnel	4	10.250 (1.258)		5.00 (0.816)		1.75 (0.500)		3.50 (0.577)	
	Retired employee	70	9.457 (1.390)		4.61 (0.873)		1.77 (0.456)		3.07 (0.804)	
	Catering	1	8.000 (-)		5.00 (-)		0.00 (-)		3.00 (-)	
	Self-employed	33	8.879 (2.497)		4.45 (1.371)		1.67 (0.645)		2.76 (0.969)	
	Homemaker	21	9.048 (2.179)		4.48 (1.030)		1.67 (0.658)		2.90 (0.995)	
	Unemployed youth	213	9.066 (1.922)		4.55 (1.052)		1.78 (0.552)		2.73 (0.995)	
	Unwilling to answer	20	8.800 (1.735)		4.50 (1.051)		1.80 (0.523)		2.50 (1.000)	
	Others	2	8.000 (1.414)		4.00 (0.000)		2.00 (0.000)		2.00 (1.414)	
	Total	578	9.228 (1.721)		4.53 (1.011)		1.80 (0.512)		2.90 (0.923)	

### 3.5. Assessment of Overall Score Concerning Mosquito

In general, the overall mean KEP score was 9.23 ± 1.72, which indicated that, on an average, the respondent knew the correct answer to 76.9% of the questions. There were marked differences in the overall score among households (general linear models functions) (*P* < 0.01). The household with the highest overall score was non-native.

### 3.6. Correlation among Knowledge, Experience, Prevention and Control Practice towards Mosquitoes

Table 4 reveals the correlation between the knowledge, experience and practices scores. The correlation analysis shows a significant positive linear correlation between knowledge and experience scores (*r* *=* 0.157*, p* *<* 0.01), knowledge and practice scores (*r* *=* 0.221*, p* *<* 0.01), and experience and practice scores (*r* *=* 0.266*, p* *<* 0.01).

**Table 4:** Correlation among knowledge, experience and practice scores.

Variable	Correlation Coefficient	*P*-Value *
Knowledge & Experience	*r* = 0.157	*P* < 0.01
Experience & Practice	*r* = 0.221	*P* < 0.01
Knowledge & Practice	*r* = 0.266	*P* < 0.01

* Correlation significant at 0.01 level (2-tailed).

## 4. Discussion

The analysis of baseline knowledge level, experience, control and preventive practices (KEP) of mosquitoes in urban Lhasa [1,20,41] should help in the development and improvement of targeted mosquito and mosquito-borne disease control and prevention strategies and measures in the future. However, prior to the present study, no information was available concerning urban Lhasa neighborhoods’ mosquito KEP levels.

Although the majority of participants had some knowledge and experience with mosquitoes, up to 6.1% stated that they cannot distinguish them from other insects, and 2.2% have no knowledge of mosquitoes. There are at least two possible reasons for this knowledge gap. First, no official documentation concerning the recent establishment of mosquitoes in Lhasa existed prior to 2013 [42]. Second, there has been insufficient health education regarding mosquitoes and their related diseases. In the present study, 26.1% of respondents indicated that they don’t have access to channels to get mosquito-related knowledge. However, they expressed their desire to gain some knowledge about the subject. These findings indicate that there is an urgent need to increase the knowledge level, broaden the channels of information, and ensure the implementation of mosquitoes prevention and control measures because of the recent presence of mosquitoes in this high altitude region [7,43,44].

The study revealed that the mean score for knowledge was higher (4.53 ± 1.01, six questions). Household register was significantly associated with knowledge score and household register with the highest score was non-native. No marked differences in human-mosquito contact frequency existed between native and non-native residents, because mosquitoes have appeared in Lhasa in recent years, but there is a possibility of differences in a matters of mosquito contact time and period of residence in the city. Questionnaire analysis showed that most of non-native registered households were a floating population. Non-Tibetan migration to Tibet had been concentrated in urban areas [45,46]. Generally, mosquito contact time of non-natives ocurred earlier than for native households while the period of residence of non-natives was shorter than that of native residents. It is probable that most non-natives had already acquired some mosquito knowledge and experience before they moved to Lhasa.

This study found a low awareness rate regarding the question “Can mosquitoes be found in varieties of water bodies?” In this case, 40.5% of the participants answered “Yes”. This rate may be due to the lack of knowledge of the general population in urban Lhasa of the breeding habits of mosquitoes.

Questionnaire analysis also revealed that mosquitoes had exerted some adverse effects on citizen’s daily life. In this study, a small percentage of local citizens went to the hospital for treatment for relatively serious complications caused by mosquito bites. The reasons why these complications were more severe in Lhasa residents may be explained as follows: first, citizens in Lhasa may be susceptible to mosquito bites due to the recent growth of mosquito population [42]. In addition, there are differences in the immune systems of people, and after being bitten, 15.2 percent of respondents did not take any kind of palliative measures. In general, mosquito bites have become an increasingly common phenomenon in Lhasa and should be given close attention in the near future.

Observations regarding mosquito experiences showed that most respondents (94.5%) had seen mosquitoes in urban Lhasa. A substantial number of them (85.3%) had been bitten by a mosquito, and more than half of these bites occurred after 2009. The study revealed that females had relatively higher experience scores than males. The possible reason might be related to differences in contact opportunity with mosquitoes and self-protection awareness. In China, the *Culex* *pipiens* complex subspecies tend to be anthropophilous [8] and these subspecies are usually found resting in dark places inside houses. According to field investigations, the majority of females recalled that their length of stay inside houses were longer than those reported by males. This surely increased the mosquito contact opportunity which may contribute to the relatively higher experience scores. In addition, it seemed that females were perceived to pay more attention to mosquito bites than males and this phenomenon needs to be further verified in future studies. In this study, the monthly income level with the highest experience score was 1000~3000 RMB. Compared with the relatively lower income group (lower than 1000 RMB), respondents in this income range tended to pay more attention to mosquito bites in the past. However, the living conditions of this group may not as good as the high income level group (higher than 3000 RMB). Therefore, they have higher experience scores than people with monthly income higher than 3000 RMB. Based on literature review, subspecies of the *Culex pipiens* complex tend to breed in polluted water bodies containing organic matter. Usually, the living areas of people with medium and low income are in locations which are close to these adverse environmental conditions and thus generate higher contact opportunities.

Based on the study, the mean score for “practices” was 2.90 ± 0.92. Household register was significantly associated with control and prevention’s score practices. The differences in this score between native and non-native people may be due to differences in mosquito-related knowledge, mosquito experience in the past, and religious doctrine. Generally speaking, registered non-natives had relatively higher mosquito-related knowledge, richer mosquito experience, and thus had higher practice scores. Interestingly, we cannot ignore the role of religious habits. Most residents of Lhasa’s sub-districts belong to the Zang (Tibetan) ethnicity.

Based on questionnaire analysis, most respondents (91.9%) believed it is necessary to control mosquitoes during the summer. Though 77.2% respondents had already taken some anti-mosquito measures, however, 22.8% of them still had not adopted any kind of anti-mosquito measures. This may represents a potential public health issue if these mosquitoes were carrying some kinds of mosquito transmitted pathogen such as West Nile virus, *etc*. The gap may relate to the several factors, including the differences in self-protection awareness, unavailability of anti-mosquito products, religious beliefs, and income levels, *etc.* Interestingly, Tibetan religious beliefs prevent them from killing mosquitoes. Therefore, a small proportion of Tibetans of devout faith did not adopt any kind of anti-mosquito measures. In this study, only a low proportion of respondents (33.4%) put forward some suggestions concerning mosquito control. Robust health education is needed to further improve local resident’s self-protection awareness and utilization of anti-mosquito products.

Based on our comprehensive analysis of the relationship between overall score and demographic background, we found that only household register was significantly associated with overall score and the household with the highest overall score was non-native. Possible reasons of the higher overall score of registered non-natives can be attributed to relatively higher knowledge and practice level, and free religious doctrine, among other factors.

Pearson correlation analysis revealed significant positive linear correlations among knowledge, experience, and practices scores. High level of mosquito-related knowledge may result from abundant mosquito experience in the past, and high level of mosquito-related knowledge help to implement mosquito control and prevention practices. In addition, enriching experience may also help adoption of more targeted practices in the future [21]. The findings are consistent with the results presented by Singh *et al.* in 2010 [47].

Great attention should be paid to the impact of some natural and social factors on the biology and ecology aspects of mosquitoes. In recent years, climate change has played an important part in the establishment of the mosquito population in urban Lhasa [48,49,50,51,52,53]. Tibet is particularly sensitive to climate change [19,54,55]. From 1961 to 2000, the greatest increase in daily mean temperatures during summer of Tibet occurred in Lhasa [56,57]. In 2009, Tibet experienced unusually warm conditions and the maximum temperature in Lhasa reached 30.4 °C, higher than the previously reported record (29.9 °C in 1971). Public reports of mosquitoes coincided with the warmest summer in Lhasa on record. These conditions mentioned above raise the risk of mosquito-borne diseases surge and outbreaks in the future [58,59,60]. Therefore, targeted health education concerning mosquito and mosquito-borne diseases are needed to improve local resident’s awareness and self-protection.

Like other retrospective surveys, recall bias might exert some impact on the information collection of questionnaire, especially past experience with mosquitoes. In addition, more questions towards mosquito experience could be added to similar studies in the future.

## 5. Conclusions

In conclusion, overall knowledge, experience, and practice levels related to mosquitoes is relatively high in urban Lhasa. Regarding future mosquito prevention and control strategies and measures, we must focus on health education of the whole population and health protection of the high risk population in Lhasa. Giving health protection to the population of native residence, especially females with monthly income between 1000 and 3000 RMB should play an important role in the prevention and control of mosquitoes and potential mosquito-borne diseases in Lhasa in the future. The findings also have guidance significance in the surveillance, control and prevention of mosquito and mosquito-borne diseases in similar altitude, latitude and longitude regions worldwide.

## Abbreviations

TAR: the Tibet Autonomous Region
KAP: Knowledge, Attitude, and Practice
KEP: Knowledge, Experience, and Practice
